# Activity of Cefepime-Zidebactam against Multidrug-Resistant (MDR) Gram-Negative Pathogens

**DOI:** 10.3390/antibiotics8010032

**Published:** 2019-03-23

**Authors:** Kenneth S. Thomson, Sameh AbdelGhani, James W. Snyder, Gina K. Thomson

**Affiliations:** 1 Department of Pathology and Laboratory Medicine, School of Medicine, University of Louisville, Louisville, KY 40202, USA; sameh.abdelghani@louisville.edu (S.A.); james.snyder@louisville.edu (J.W.S.); ginamu@ulh.org (G.K.T.); 2 Faculty of Pharmacy, Beni-Suef University, Beni-Suef 62511, Egypt; 3 Microbiology Department, University of Louisville Hospital, Louisville, KY 40202, USA

**Keywords:** carbapenemase-producing organism, carbapenemase, zidebactam, therapy

## Abstract

This study compared the activity of cefepime + zidebactam (FEP-ZID) and selected currently available antibacterial agents against a panel of multidrug-resistant (MDR) clinical isolates chosen to provide an extreme challenge for antibacterial activity. FEP–ZID had a very broad and potent in vitro spectrum of activity, and was highly active against many MDR isolates of *Enterobacterales*, *Pseudomonas aeruginosa*, and *Acinetobacter baumannii*. Notably, it inhibited isolates producing carbapenemases of Ambler classes A, B, and D, and *P. aeruginosa* isolates with multiple resistance mechanisms including combinations of upregulated efflux, diminished or non-functional OprD porins, and AmpC overproduction. Its clinical role will be determined initially by the breakpoints assigned to it, comparison studies with other investigational β-lactamase inhibitor combinations, and ultimately by the developing body of therapeutic outcome data.

## 1. Introduction

Gram-negative multidrug-resistant (MDR) pathogens, particularly carbapenemase-producing organisms (CPOs), cause infections of high mortality that are typically treated with antibiotic combinations that include toxic drugs such as polymyxins and aminoglycosides [1]. Recent Food and Drug Administration (FDA) approvals of the β-lactamase inhibitor combinations ceftazidime + avibactam and meropenem + vaborbactam have created the potential for safer treatments of infections by CPOs producing Ambler class A carbapenemases, and also pathogens that produce AmpC and/or extended spectrum β-lactamases [2,3,4,5,6]. Other β-lactamase inhibitor combinations are under development, e.g., imipenem + relebactam, aztreonam + avibactam, meropenem + nacubactam, cefepime + zidebactam, and cefepime + VNRX-5133, some of which are active against CPOs producing Ambler class A, B, and D carbapenemases [2,4,5,7,8,9,10,11,12,13]. The clinical potential of the new β-lactamase inhibitor combinations is significant, but the optimism about them should be tempered by reports of reduced susceptibility or resistance to ceftazidime + avibactam in clinical isolates sometimes emerging during therapy [14,15,16,17,18,19]. Arising concerns include whether the ceftazidime + avibactam resistance can impact the effectiveness of the developmental combinations, and whether or not ceftazidime + avibactam resistance indicates the need to avoid suboptimal combinations of co-drugs and inhibitors. For example, ceftazidime was previously associated with the development of resistance when used for treating infections by extended spectrum β-lactamase (ESBL) nd AmpC producers [20,21,22]. Therefore, given the benefit of hindsight, it was a questionable choice of co-drug for avibactam. Furthermore, it is possible that avibactam, a diazobicyclooctane, has attributes that may prove to be suboptimal in that various types of β-lactamases may develop resistance to it [19,23,24], and its clinical spectrum of carbapenemase coverage is limited to class A and possibly OXA-48-like class D carbapenemases [7]. Class B carbapenemases and the class D carbapenemases of *Acinetobacter* spp. are gaps in its spectrum. It is also currently unknown if resistance to avibactam may confer resistance to its diazobicyclooctane analogues, relebactam and nacubactam.

Cefepime + zidebactam (FEP–ZID) is a unique combination agent in that its bicyclo-acyl hydrazide component, zidebactam, is a β-lactamase inhibitor which possesses intrinsic antibacterial activity. That is, zidebactam can both protect cefepime from hydrolysis by β-lactamases and extend its spectrum of antibacterial activity [12,13]. With the aim of assessing its activity against Gram-negative isolates chosen to provide an extreme challenge of antibacterial activity, a study was designed to evaluate the in vitro activity of FEP–ZID and selected currently available antibacterial agents against a panel of well-characterized MDR clinical isolates, with a special focus on diverse types of carbapenemase producers.

## 2. Methodology

### 2.1. Isolates 

The study isolates consisted of 132 isolates, comprising of *Enterobacterales* (*n* = 102), *Pseudomonas aeruginosa* (*n* = 18), and *Acinetobacter baumannii* (*n* = 12) that were previously characterized by molecular, phenotypic, and biochemical tests for types of β-lactamase production. Some *P. aeruginosa* isolates were also characterized for degree of efflux and porin expression. These isolates were donated by the Centers for Disease Control and Prevention, Food and Drug Administration Antimicrobial Resistance Isolate Bank, and the Microbiology Laboratory at the University of Louisville Hospital. The study also includes isolates from previously published studies [25,26]. The specific resistance mechanisms for the three groups of organisms (*Enterobacterales*, *P. aeruginosa*, and *A. baumannii*) are provided in Table 1 and Supplementary Tables S1 and S2 for individual isolates. The class A carbapenemases, produced by 39 isolates, included KPC-2, KPC-3, KPC-4, KPC-5, KPC-6, KPC-8, KPC-18, NMC-A, SME-type, and GES-5. The class B carbapenemases, produced by 28 isolates, included IMP-1, IMP-7, IMP-8, VIM-1, VIM-2, VIM-4, VIM-7, NDM-1, GIM-1, and SPM-1, and the class D carbapenemases, produced by 22 isolates, included OXA-23, OXA-40, OXA-58, OXA-48, OXA-181, and OXA-232. Five CPOs produced two classes of carbapenemases. ESBLs that were produced by 22 non-CPO isolates of *Enterobacteriaceae* included CTX-M-1, CTX-M-2, CTX-M-9, CTX-M-12, CTX-M-14, CTX-M-15, CTX-M-18, CTX-M-28, CTX-M-45, TEM-16, SHV-3, SHV-4, SHV-7, and SHV-12, with some isolates co-producing an AmpC β-lactamase. Ten isolates of *Enterobacterales* produced plasmid-mediated AmpCs of CMY, DHA, FOX, and LAT types. The above-mentioned β-lactamases are ones capable of causing resistance to at least one of the study drugs. Types of isolates included in the study but not included in the above groups either produced limited-spectrum β-lactamases or had other resistance mechanisms, such as reduced permeability or upregulated efflux.

### 2.2. Susceptibility Testing

Antibiotic susceptibility test results were reported as minimum inhibitory concentrations (MICs), and interpreted against breakpoints where possible. Breakpoints are specific concentrations of antibacterial agents considered to be predictive of therapeutic responses to normally attained tissue of body fluid concentrations. They are typically described as susceptible (agent is likely to be clinically active), resistant (agent is likely to be ineffective), and intermediate (activity uncertain when agent is dosed as recommended, but may be effective at higher doses). The in vitro activities of FEP–ZID, piperacillin + tazobactam, ceftolozane + tazobactam, cefepime, imipenem, ceftazidime, levofloxacin, amikacin, and polymyxin B were determined by Clinical and Laboratory Standards Institute (CLSI) agar dilution methodology, using doubling dilutions of antibacterial agents incorporated in Mueller–Hinton agar (BD Diagnostics, Sparks, MD, USA), inocula of 10^4^ colony forming units (cfu)/spot, and overnight incubation at 35 °C. Concentrations of zidebactam and β-lactamase inhibitors comprised a fixed 4 μg/mL of tazobactam for ceftolozane + tazobactam and piperacillin + tazobactam, and a 1:1 ratio combination for FEP–ZID and cefoperazone + sulbactam. MICs of β-lactamase inhibitor combinations were reported for only the β-lactam co-drug. Where applicable, susceptibility was interpreted using appropriate CLSI breakpoints for relevant organism groups [27]. The CLSI high-dose susceptible breakpoint of ≤8 μg/mL was used for cefepime when tested alone. In the absence of CLSI breakpoints, susceptibility was not interpreted for tests with FEP–ZID and cefoperazone + sulbactam. The control strains were *Escherichia coli* ATCC 25922, *E. coli* ATCC 35218, *Klebsiella pneumoniae* ATCC 700603, and *P. aeruginosa* ATCC 27853. Results were summarized as the concentrations that inhibited 50% and 90% of isolates (MIC_50_ and MIC_90_ values, respectively). Percent susceptibility was calculated only for organism groups with a minimum of 10 isolates.

The sources of the laboratory powders of antibacterial agents were: Wockhardt Research Center Aurangabad, India (cefepime, ceftolozane, cefoperazone, zidebactam, and tazobactam); Thermo Fisher Scientific Chemicals Inc., Ward Hill, MA, USA (imipenem and amikacin); TCI America, Portland, OR, USA (levofloxacin, piperacillin, and ceftazidime); and VWR International, LLC, Batavia, IL, USA (polymyxin B).

## 3. Results

### 3.1. Activity against All Isolates 

As shown in Table 2, FEP–ZID and polymyxin B were the most potent agents against this collection of predominantly carbapenemase-producing isolates. The MIC_50_ and MIC_90_ values for FEP–ZID were 0.5 and 16 μg/mL, respectively, and they were 1 and ≥16 μg/mL, respectively, for polymyxin B. With the exception of imipenem (MIC_50_ and MIC_90_ values 4 and 128 μg/mL, respectively), all other comparison agents had 32 to 256-fold higher MIC_50_ values than FEP–ZID and high off-scale MIC_90_ values.

### 3.2. Activity against CPOs 

In tests with all CPOs, FEP–ZID was the most potent agent, with respective MIC_50_ and MIC_90_ values of 1 and 16 μg/mL. Polymyxin B was the next most potent agent, with MIC_50_ and MIC_90_ values of 1 and ≥16 μg/mL (Table 2). Against isolates producing class A carbapenemases (Table S1), FEP–ZID and polymyxin B had respective MIC_50_ and MIC_90_ values of 0.5 and 8 μg/mL, and 1 and ≥16 μg/mL. Cefepime (MIC_50_ and MIC_90_ values of 4 and ≥128 μg/mL, respectively) and amikacin (MIC_50_ and MIC_90_ values of 8 and 64 μg/mL, respectively) were the only other agents exhibiting activity against more than 50% of the class A CPOs (both 56.1% susceptibility). Isolates producing class B carbapenemases were more resistant, but FEP–ZID (MIC_50_ and MIC_90_ values of 2 and 32 μg/mL, respectively) was distinctly more active than the comparison agents, all of which had high, off-scale MIC_90_ values, with very few isolates being susceptible. Similarly, against isolates producing class D carbapenemases, with the exception of polymyxin B, FEP–ZID, with MIC_50_ and MIC_90_ values of 2 and 16 μg/mL, respectively, was distinctly more active than the comparison agents. With the exception of one isolate of OXA-48-producing *K. pneumoniae* (MIC ≥ 128 μg/mL), all other *Enterobacterales* producing OXA-48-like carbapenemases had FEP–ZID MICs of ≤2 μg/mL. OXA-carbapenemase-producing isolates of *A. baumannii* were more resistant overall than OXA-producing *Enterobacterales*. *A. baumannii* isolates producing OXA-23 or OXA-40 had FEP–ZID MICs in the range 4 to 32 μg/mL. One isolate producing two carbapenemases (OXA-23 + NDM) had a FEP–ZID MIC of ≥128 μg/mL. All other dual carbapenemase producers had FEP–ZID MICs of ≤2 μg/mL.

### 3.3. Activity against ESBL-Producing and Plasmid-Mediated AmpC-Producing Enterobacterales

FEP–ZID, polymyxin B, and imipenem were the most active agents against ESBL-producing *Enterobacterales*. Only two agents, FEP–ZID and polymyxin B, had low MIC_90_ values (2 μg/mL for both agents). Imipenem was the next most active agent (MIC_90_ value of 16 μg/mL and 83.4% susceptibility). All other comparison agents had high, off-scale MIC_90_ values. Among these, only amikacin (72.7%), ceftolozane + tazobactam (59.1%), and piperacillin + tazobactam (59.1%) inhibited more than 50% of the ESBL producers.

Against the plasmid-mediated AmpC-producing *Enterobacterales*, FEP–ZID (MIC_50_ and MIC_90_ values of 0.12 and 2 μg/mL, respectively) and cefepime (MIC_50_ and MIC_90_ values of 0.5 and 8 μg/mL, respectively, and 90% susceptibility) were the most active agents. Polymyxin B was the next most active agent (MIC_50_ and MIC_90_ values were both 1 μg/mL). Eight of the 10 plasmid-mediated AmpC-producing isolates had FEP–ZID MICs of ≤0.25 μg/mL. The agents most compromised by plasmid-mediated AmpCs were ceftolozane + tazobactam, piperacillin + tazobactam, and levofloxacin. 

### 3.4. Activity against MDR Enterobacterales, P. aeruginosa, and A. baumannii Irrespective of Resistance Mechanisms 

FEP–ZID was the most active agent against all *Enterobacterales*, with a dramatically lower MIC_90_ value (2 μg/mL) than the other study drugs (128 μg/mL or high, off-scale values). Of the drugs for which breakpoints were available, the most active were amikacin (58.8% susceptible) and imipenem (47.1% susceptible). 

Against all *P. aeruginosa* isolates, polymyxin B was the most potent agent, with MIC_50_ and MIC_90_ values of 2 and 4 μg/mL, respectively, followed by FEP–ZID (MIC_50_ and MIC_90_ values of 8 and 32 μg/mL, respectively). With the exceptions of amikacin and polymyxin B (both 61.1% susceptibility), the other agents inhibited less than 40% of the isolates at the CLSI susceptible breakpoints. Agents with most potency against the more susceptible *P. aeruginosa* isolates, indicated by MIC_50_ values, were polymyxin B (2 μg/mL), FEP–ZID (8 μg/mL), ceftolozane + tazobactam (16 μg/mL), and amikacin (16 μg/mL). Reduced susceptibility to FEP–ZID was either associated with IMP or VIM class B carbapenemases, or combinations of mechanisms such as overexpressed MexAB-OprM or MexXY efflux, diminished OprD function, and high-level AmpC production. 

Polymyxin B (MIC_50_ and MIC_90_ values of 1 and 2 μg/mL, respectively, and 91.2% susceptibility) and FEP–ZID (MIC_50_ and MIC_90_ values of 16 and 32 μg/mL, respectively) were the most active agents against *A. baumannii*. On the basis of CLSI breakpoints, amikacin (50.0% susceptibility) was the next most active agent, with the other drugs with breakpoints having susceptibility rates of only 0% to 8.3%. 

## 4. Discussion

This study comprised a very challenging panel of isolates. The diversity of the range of single-carbapenemase and dual-carbapenemase production provided the strongest challenge for FEP–ZID activity in this study, and thereby extended our understanding of the activity of FEP–ZID and provided insights into its potential against MDR isolates of *Enterobacterales*, *P. aeruginosa*, and *A. baumannii*. FEP–ZID was notable for its activity against CPOs producing all three Ambler classes of carbapenemases and against *P. aeruginosa* with multiple resistance mechanisms, including combinations of upregulated efflux, diminished or non-functional OprD porins, and AmpC overproduction. Especially notable was the ability of zidebactam to enhance the activity of cefepime against CPOs producing class D carbapenemases. Nontoxic treatment for infections by MDR *A. baumannii* isolates producing OXA class D carbapenemases is currently an unmet medical need. There is also a need for effective therapies for infections by MDR isolates of *P. aeruginosa* [28,29]. The activity of FEP–ZID against such isolates warrants clinical investigation.

If the potency of FEP–ZID translates into clinical efficacy in humans, FEP–ZID could be considered as an expanded spectrum β-lactam/β-lactam enhancer combination. This is consistent with previous reports [12,13,30]. The need for FEP–ZID breakpoints and recommended testing methods is urgent, because the CPO problem is global and associated with high mortality [31,32,33,34,35]. There are no drugs to treat all CPO infections, and resistance continues to emerge. It is essential to reduce the mortality and control the spread of these organisms, and it must be done now. The need for effective therapies is urgent. To avoid delays from hindering the quest for effective therapy of all types of CPO infections, recent surveillance data, population pharmacokinetic (PK), Monte Carlo-based pharmacokinetic/pharmacodynamic (PK/PD) studies, and animal models utilizing human-simulated dosing regimens against high MIC strains can be analyzed to expedite breakpoint and testing recommendations. 

At this time, in the absence of FEP–ZID breakpoints, we avoided making interpretations of susceptibility or resistance in our analysis, and solely reported in vitro potency data for this agent. 

## 5. Conclusions

In conclusion, FEP–ZID exhibited broad spectrum potency against a wide range of MDR Gram-negative pathogens in this study. Depending on the breakpoints that are assigned, its antibacterial spectrum may provide more comprehensive CPO coverage for empirical therapy of infections than the current limited-spectrum agents, ceftazidime + avibactam and meropenem + vaborbactam. The clinical role of FEP–ZID will be determined initially by the breakpoints assigned to it, followed by comparison studies with other investigational β-lactamase inhibitor combinations and, ultimately, by the developing body of therapeutic outcome data.

## Figures and Tables

**Table 1:** Resistance mechanisms of organism groups.

Organism Group	Type of Resistance Mechanism	Specific Mechanisms
*Enterobacterales*	Class A Carbapenemase	KPC-2, KPC-3, KPC-4, KPC-6, KPC-8, KPC-18, NMC-A, SME-type, GES-5
	Class B Carbapenemase	IMP-1, IMP-8, VIM, VIM-1, VIM-2, NDM, NDM-1,
	Class D Carbapenemase	OXA-48, OXA-181, OXA-232
	Dual Carbapenemase Classes	KPC-18 + VIM-1, OXA-181 + NDM, NDM-1 + OXA-181+ CTX-M-15
	ESBL (+/- AmpC)	CTX-M-1, CTX-M-2, CTX-M-9, CTX-M-12, CTX-M-14, CTX-M-15, CTX-M-18, CTX-M-28, CTX-M-45, TEM-16, SHV-3, SHV-4, SHV-7, SHV-12
	Plasmid-mediated AmpC	CMY, DHA, FOX, LAT
*P. aeruginosa*	Class A Carbapenemase	KPC, KPC-5,
	Class B Carbapenemase	IMP-1, IMP-7, IMP-18, VIM-2, VIM-4, VIM-7, GIM-1, SPM-1
	Efflux/Porin/AmpC/ESBL	Upregulation for MexAB-OprM, MexEF-OprN, MexXY-OprM, diminished or nonfunctional OprD, AmpC upregulation, OXA-45 ESBL
*A. baumannii*	Class A Carbapenemase	KPC
	Class B Carbapenemase	NDM
	Class D Carbapenemase	OXA-23, OXA-40, OXA-58
	Dual Carbapenemase Classes	OXA-23 + NDM

**Table 2:** Potencies against all isolates.

Agent	Results Against All 132 Isolates in μg/mL		
	MIC Range	MIC50	MIC90
FEP-ZID	0.03 – ≥128	0.5	16
Cefepime	0.03 – ≥128	16	≥128
Cefoperazone + sulbactam	≤0.25 – ≥64	16	≥64
Ceftolozane + tazobactam	≤0.06 – ≥64	32	≥64
Piperacillin + tazobactam	0.5 – ≥256	128	≥256
Ceftazidime	≤0.06 – ≥256	128	≥256
Imipenem	≤0.06 – ≥256	4	128
Amikacin	≤0.25 – ≥256	16	≥256
Levofloxacin	0.03 – ≥32	16	≥32
Polymyxin B	0.25 – ≥16	1	≥16

## Supplementary Materials

The following are available online at https://www.mdpi.com/2079-6382/8/1/32/s1, Table S1: Activity of FEP-ZID and Comparison Agents, Table S2: MIC of FEP-ZID and Comparators against All Tested Strains.
