# Supplementary material for: Activity of Cefepime-Zidebactam against Multidrug-Resistant (MDR) Gram-Negative Pathogens
Source: Antibiotics (Basel). 2019 Mar 23;8(1):32. doi: 10.3390/antibiotics8010032 (PMC6466586; doi:10.3390/antibiotics8010032)
Supplement: Supplementary file 1 [file antibiotics-08-00032-s001.pdf]

Supplemental Material

MIC (µg/mL) of FEP-ZID and Comparators against All Tested Strains

(FEP: Cefepime; ZID: Zidebactam; CFP: cefoperazone; SUL: Sulbactam; TOL: Ceftolozane; PIP: Piperacillin; TAZ: Tazobactam; CAZ: ceftazidime, IPM: Imipenem; AMIK: Amikacin; LEV: Levofloxacin; PB: Polymyxin B)

Isolate	Organism	Resistance Mechanism(s)	FEP	FEP-ZID	CFP-SUL	TOL-TAZ	PIP-TAZ	CAZ	IPM	AMIK	LEV	PB
G1646	*E. coli*	CTX-M-1	4	0.12	8	0.5	1	4	0.25	1	0.5	0.5
G1647	*E. coli*	CTX-M-45	≥128	0.12	16	1	16	8	0.12	2	≥32	0.5
G1655	*E. coli*	CTX-M-14	4	0.12	4	0.12	2	1	0.12	≥256	0.06	1
G1659	*E. coli*	SHV-3	16	0.25	1	0.5	4	≥256	0.5	4	0.5	0.5
G1661	*E. coli*	CTX-M-15, OXA-1	32	0.25	4	0.25	16	64	0.12	16	16	0.5
G1671	*E. coli*	CTX-M-14-like	4	0.12	8	0.5	2	2	0.12	8	16	0.5
G1681	*E. coli*	CTX-M-18	16	0.12	4	0.5	4	4	0.12	64	0.12	0.5
G1633	*E. coli*	SHV-3	0.12	0.06	0.5	≤0.06	1	2	0.12	4	0.03	0.5
0086	*E. coli*	CTX-M-9	64	0.5	32	0.5	16	4	0.12	4	16	0.5
G1743	*E. coli*	CTX-M-12	32	0.12	16	2	8	32	0.12	8	0.06	2
0081	*E. coli*	CMY-2 type	0.5	0.06	4	1	8	32	0.12	4	≥32	1
0085	*E. coli*	CMY-2	1	0.25	8	8	32	64	0.25	2	≥32	1
G1683	*E. coli*	DHA-like	0.12	0.25	2	0.5	4	64	4	4	≥32	1
G164	*E. coli*	High AmpC, SHV-12 like	16	2	16	16	128	≥256	16	16	≥32	1
G15238	*E. coli*	KPC-3 like	16	0.25	32	16	≥256	128	4	2	≥32	0.5
G15239	*E. coli*	KPC-3 like	2	2	8	8	128	64	2	16	≥32	0.5
G1736	*E. coli*	GES-5, TEM-1 like, OXA-1, CTX-M	≥128	0.5	32	8	64	≥256	0.12	≥256	≥32	0.5
0048	*E. coli*	NDM	≥128	0.5	≥64	≥64	≥256	≥256	4	≥256	≥32	0.5

Isolate	Organism	Resistance Mechanism(s)	FEP	FEP-ZID	CFP-SUL	TOL-TAZ	PIP-TAZ	CAZ	IPM	AMIK	LEV	PB
0042	*K. pneumoniae*	CTX-M28, OMPK36, OMPK-35	≥128	1	32	≥64	≥256	128	≤0.06	8	≥32	1
0043	*K. pneumoniae*	SHV-12, OMPK-36	32	1	8	16	≥256	≥256	≤0.06	2	4	1
0044	*K. pneumoniae*	TEM-1, SHV-1, CTX-M-15	≥128	4	32	≥64	≥256	≥256	≤0.06	64	≥32	1
0107	*K. pneumoniae*	CTX-M2, OMPK-36	≥128	2	≥64	≥64	≥256	64	2	≥256	≥32	1
0109	*K. pneumoniae*	TEM-1, SHV-1, CTX-M-15	≥128	4	≥64	≥64	≥256	≥256	0.5	32	≥32	≥16
G1660	*K. pneumoniae*	TEM-16	2	0.25	8	32	≥256	≥256	0.12	16	2	1
0079	*K. pneumoniae*	CTX-M14,DHA-1,OMPK35	≥128	2	≥64	32	≥256	32	32	≥256	≥32	1
G1616	*K. pneumoniae*	LAT-4	1	0.25	4	16	64	≥256	0.25	16	0.06	2
G162	*K. pneumoniae*	FOX-5, SHV-4 like	0.25	0.12	4	2	16	64	0.12	1	0.12	1
G1685	*K. pneumoniae*	CMY-2-like	8	2	≥64	32	≥256	≥256	16	1	2	1
G1702	*K. pneumoniae*	FOX-1	0.12	0.06	4	0.5	4	32	0.12	8	0.06	1
G1754	*K. pneumoniae*	TEM-1,SHV-1, OXA-9, SHV-5, ACT-1	0.5	0.12	8	16	≥256	≥256	≤0.06	64	0.25	1
G15007	*K. pneumoniae*	KPC-2-like	1	0.12	8	16	64	64	0.25	32	≥32	1
G1510	*K. pneumoniae*	KPC-4	64	0.5	32	32	128	≥256	0.5	1	≥32	1
G1511	*K. pneumoniae*	KPC-4	4	0.12	8	32	64	≥256	0.5	1	≥32	1
G1513	*K. pneumoniae*	KPC-3 like	8	0.12	16	32	128	≥256	0.25	64	≥32	1

Isolate	Organism	Resistance Mechanism(s)	FEP	FEP-ZID	CFP-SUL	TOL-TAZ	PIP-TAZ	CAZ	IPM	AMIK	LEV	PB
G1514	*K. pneumoniae*	KPC-3,SHV-12-like,SHV-1-like,OXA-9-like,TEM-1-like	2	0.12	8	16	128	≥256	0.25	64	≥32	1
G15313	*K. pneumoniae*	KPC-2-like	2	0.12	8	16	128	64	≤0.06	64	≥32	1
G157	*K. pneumoniae*	KPC-8	≥128	2	≥64	≥64	≥256	≥256	32	64	≥32	1
G158	*K. pneumoniae*	KPC-8	≥128	2	≥64	≥64	≥256	≥256	32	64	≥32	1
G159	*K. pneumoniae*	KPC-6	2	0.25	16	32	≥256	128	2	≤0.25	16	1
G1639	*K. pneumoniae*	KPC-2	8	0.5	32	16	≥256	64	8	2	0.12	1
G1668	*K. pneumoniae*	KPC	32	0.5	≥64	≥64	≥256	≥256	8	8	≥32	1
G1675	*K. pneumoniae*	KPC-2, SHV-5-like,TEM-1-like	≥128	1	≥64	32	≥256	≥256	32	32	≥32	1
G1725	*K. pneumoniae*	KPC	64	1	≥64	≥64	≥256	≥256	64	64	≥32	≥16
G1729	*K. pneumoniae*	KPC	4	0.25	16	8	≥256	32	8	64	0.12	1
0034	*K. pneumoniae*	IMP	2	0.12	16	≥64	8	128	0.5	2	1	1
0040	*K. pneumoniae*	VIM	≥128	1	≥64	≥64	≥256	≥256	64	64	≥32	≥16
0041	*K. pneumoniae*	NDM	64	0.5	≥64	≥64	≥256	≥256	4	≥256	16	1
0046	*K. pneumoniae*	VIM	≥128	1	≥64	≥64	≥256	≥256	32	64	≥32	≥16
0076	*K. pneumoniae*	VIM	8	0.12	32	≥64	128	≥256	4	16	1	1
0080	*K. pneumoniae*	IMP	0.03	0.06	≤0.25	0.25	4	0.5	0.5	2	0.25	1
G15291	*K. pneumoniae*	VIM-like,SHV-12-like,SHV-1-like	≥128	32	≥64	≥64	≥256	≥256	32	64	≥32	≥16
G15300	*K. pneumoniae*	IMP-8	8	0.12	16	≥64	8	≥256	1	32	≥32	≥16
G15353	*K. pneumoniae*	NDM-1,CTX-M-15-like,SHV-1-like	≥128	0.5	≥64	≥64	≥256	≥256	16	≥256	32	1
G15409	*K. pneumoniae*	NDM-1,CTX-M-15,TEM-1,OXA-1	32	0.25	≥64	≥64	≥256	≥256	16	32	≥32	1

Isolate	Organism	Resistance Mechanism(s)	FEP	FEP-ZID	CFP-SUL	TOL-TAZ	PIP-TAZ	CAZ	IPM	AMIK	LEV	PB
G15406	*K. pneumoniae*	NDM-1, CTX-M-15,OXA-181	≥128	1	≥64	≥64	≥256	≥256	128	16	≥32	1
0075	*K. pneumoniae*	OXA-232	≥128	1	32	≥64	≥256	128	1	≥256	≥32	1
G15480	*K. pneumoniae*	OXA-48	0.06	0.06	4	0.5	128	0.5	1	1	1	1
G15481	*K. pneumoniae*	OXA-48	≥128	2	32	≥64	≥256	128	8	16	≥32	1
G15482	*K. pneumoniae*	OXA-48	≥128	≥128	32	≥64	≥256	≥256	1	32	≥32	≥16
G15483	*K. pneumoniae*	OXA-48	32	0.5	16	32	≥256	128	0.5	16	8	2
G15484	*K. pneumoniae*	OXA-48	≥128	2	≥64	≥64	≥256	≥256	4	32	≥32	1
G15485	*K. pneumoniae*	OXA-48	≥128	2	≥64	≥64	≥256	≥256	2	≥256	≥32	1
G15486	*K. pneumoniae*	OXA-48	≥128	2	≥64	≥64	≥256	≥256	2	8	≥32	≥16
G15489	*K. pneumoniae*	OXA-48	≥128	0.5	16	≥64	≥256	≥256	2	32	≥32	8
G15490	*K. pneumoniae*	OXA-48	32	0.5	16	≥64	≥256	128	1	4	≥32	0.5
G15491	*K. pneumoniae*	OXA-181	≥128	2	≥64	≥64	≥256	≥256	64	16	≥32	1
G163	*K. oxytoca*	DHA-like, SHV ESBL, TEM-1-like, K1	0.12	0.06	2	0.25	4	4	0.5	8	4	1
G15004	*K. oxytoca*	KPC	1	0.5	≥64	32	≥256	≥256	0.25	32	4	0.5
G1640	*K. oxytoca*	KPC	64	2	≥64	≥64	≥256	≥256	64	64	4	0.5
G166	*E. aerogenes*	SHV-4,TEM-1	0.5	0.12	1	0.5	4	64	0.5	2	0.06	1
G1638	*E. aerogenes*	High AmpC,SHV-5-like	0.5	0.06	1	0.25	8	64	0.5	2	0.06	1
G329	*E. aerogenes*	Hi AmpC, SHV-5-like	0.5	0.12	1	0.25	4	64	0.5	2	0.06	1
0074	*E. aerogenes*	OXA-48	0.25	0.12	8	1	≥256	1	1	4	0.25	1
0065	*E. cloacae*	cAmpC	≥128	2	≥64	≥64	≥256	≥256	2	16	≥32	1
G15162	*E. cloacae*	NMC-A	0.03	0.03	≤0.25	0.25	4	0.5	64	2	0.06	≥16
G152	*E. cloacae*	KPC	2	0.25	16	4	128	8	0.5	≤0.25	0.25	1

Isolate	Organism	Resistance Mechanism(s)	FEP	FEP-ZID	CFP-SUL	TOL-TAZ	PIP-TAZ	CAZ	IPM	AMIK	LEV	PB
G15270	*E. cloacae*	KPC	16	0.25	16	16	128	≥256	4	4	1	0.5
G15296	*E. cloacae*	KPC	4	0.25	32	8	128	32	4	8	0.12	0.5
G15306	*E. cloacae*	KPC-2-like	2	1	32	16	128	128	≤0.06	2	2	1
G15315	*E. cloacae*	KPC-2-like	4	0.5	32	8	128	32	8	2	8	1
G15333	*E. cloacae*	KPC-2-like	4	0.12	8	8	128	16	4	2	0.06	1
G15405	*E. cloacae*	NDM	64	0.25	≥64	≥64	≥256	≥256	4	≥256	≥32	1
G1691	*E. cloacae*	IMP-8	2	0.12	2	32	2	64	2	32	0.5	≥16
0038	*E. cloacae*	NDM	≥128	2	≥64	≥64	≥256	≥256	≥256	≥256	≥32	1
G6809	*E. cloacae*	KPC-18,VIM-1	32	0.25	≥64	≥64	≥256	≥256	4	16	2	1
G6810	*E. cloacae*	KPC-18,VIM-1	64	0.5	≥64	≥64	≥256	≥256	4	32	2	1
0066	*E. cloacae*	OXA-232	≥128	2	≥64	≥64	≥256	≥256	2	16	≥32	1
G1749	*C. freundii*	KPC	4	0.12	32	8	128	32	0.5	2	≥32	1
G15327	*C. freundii complex*	KPC-3 like	1	0.12	16	4	128	4	1	≤0.25	≥32	1
0057	*M. morganii*	NDM	8	2	≥64	≥64	16	≥256	8	4	16	≥16
G1649	*S. marcescens*	SHV-7	1	0.5	8	4	32	64	0.12	1	≥32	≥16
G1764	*S. marcescens*	SME-like	16	0.06	1	0.5	4	0.25	64	≥256	0.25	8
0091	*S. marcescens*	SME	0.06	2	0.5	0.5	1	≤0.06	128	1	0.25	≥16
0099	*S. marcescens*	SME	0.06	1	1	0.5	2	≤0.06	64	1	0.25	≥16
G15081	*S. marcescens*	SME-like	0.03	0.25	≤0.25	0.5	1	≤0.06	64	1	0.06	≥16
G15166	*S. marcescens*	SME-like	0.06	0.12	1	0.5	2	≤0.06	64	1	0.12	≥16
G153	*S. marcescens*	KPC	16	1	32	32	128	64	32	2	4	≥16
G15126	*S. marcescens*	IMP-1	64	16	≥64	≥64	64	≥256	128	4	16	2
G15407	*S. marcescens*	NDM-1	64	16	32	≥64	32	≥256	128	≥256	8	2
G15408	*P. rettgeri*	NDM-1	≥128	≥128	≥64	≥64	≥256	≥256	128	≥256	≥32	≥16
G1748	*H. alvei*	KPC	1	0.5	8	8	64	64	4	2	0.25	≥16

**Isolate**	**Organism**	**Resistance Mechanism(s)**	**FEP**	**FEP-ZID**	**CFP-SUL**	**TOL-TAZ**	**PIP-TAZ**	**CAZ**	**IPM**	**AMIK**	**LEV**	**PB**
G16288	*P. aeruginosa*	Overexpressed MexXY, OprD not functional	4	4	4	0.5	8	4	16	8	8	2
G16291	*P. aeruginosa*	Overexpressed mexXY, OprD diminished, derepressed AmpC	16	16	32	2	128	32	16	16	16	2
G16350	*P. aeruginosa*	Overexpressed MexAB-OprM, derepressed AmpC	NG	NG	NG	NG	NG	NG	NG	NG	NG	NG
G16352	*P. aeruginosa*	Overexpressed MexAB-OprM, OprD diminished, derepressed AmpC	32	32	≥64	1	64	32	≥256	16	8	2
G16368	*P. aeruginosa*	Overexpressed MexAB-OprM, OprD diminished	8	4	8	0.5	16	8	32	4	4	2
G16369	*P. aeruginosa*	Overexpressed MexEF-OprM, OprD diminished	16	2	1	1	2	4	≥256	≥256	≥32	4
G1778	*P. aeruginosa*	Partially derepressed	0.5	0.5	≤0.25	0.5	0.5	4	2	4	0.25	2
G15	*P. aeruginosa*	KPC-5	≥128	8	≥64	16	≥256	128	≥256	16	≥32	2
G15013	*P. aeruginosa*	KPC	≥128	8	≥64	16	≥256	64	≥256	32	≥32	2

Isolate	Organism	Resistance Mechanism(s)	FEP	FEP-ZID	CFP-SUL	TOL-TAZ	PIP-TAZ	CAZ	IPM	AMIK	LEV	PB
G15311	*P. aeruginosa*	KPC	≥128	8	≥64	16	≥256	128	≥256	16	≥32	4
0054	*P. aeruginosa*	VIM-4	64	8	≥64	≥64	128	64	≥256	2	≥32	2
0103	*P. aeruginosa*	IMP-1	≥128	8	≥64	≥64	16	≥256	≥256	32	≥32	4
G15015	*P. aeruginosa*	IMP-7	≥128	8	≥64	≥64	16	≥256	≥256	32	≥32	4
G15017	*P. aeruginosa*	VIM-2	32	32	≥64	≥64	128	128	128	8	≥32	2
G15020	*P. aeruginosa*	VIM-7,OXA-45	≥128	32	≥64	≥64	≥256	≥256	≥256	32	≥32	0.5
G15297	*P. aeruginosa*	IMP-18	≥128	16	≥64	≥64	128	≥256	128	128	≥32	2
G15303	*P. aeruginosa*	GIM-1	8	8	≥64	16	128	128	128	1	16	≥16
G15304	*P. aeruginosa*	SPM-1	≥128	8	≥64	≥64	128	≥256	≥256	≥256	≥32	4
G15402	*P. aeruginosa*	IMP-7	≥128	8	≥64	≥64	16	≥256	128	4	≥32	4
G1694	*A. baumannii*	KPC	≥128	32	16	≥64	≥256	≥256	16	32	≥32	1
0036	*A. baumannii*	OXA-40	32	16	4	32	≥256	≥256	64	32	≥32	≥16
0045	*A. baumannii*	OXA-23	32	16	16	≥64	≥256	≥256	16	4	16	1
0063	*A. baumannii*	OXA-40	32	16	4	32	≥256	≥256	64	2	16	1
0070	*A. baumannii*	OXA 58	2	2	2	2	128	16	2	0.5	4	0.25
G1674	*A. baumannii*	OXA-23	32	8	16	≥64	≥256	≥256	16	4	2	1
G1687	*A. baumannii*	OXA-23	≥128	16	32	≥64	≥256	≥256	16	≥256	8	1
G1703	*A. baumannii*	OXA-23	32	16	16	16	≥256	128	32	≥256	16	2
G1732	*A. baumannii*	OXA-23	32	16	16	16	≥256	128	32	≥256	8	1
G1734	*A. baumannii*	OXA-40	16	4	32	8	≥256	128	128	1	8	1
G1744	*A. baumannii*	OXA-40	16	16	4	8	≥256	128	128	16	16	2
0083	*A. baumannii*	OXA-23, NDM	≥128	≥128	≥64	≥64	≥256	≥256	128	≥256	8	2
